# HSV-2-Driven Increase in the Expression of α_4_β_7_ Correlates with Increased Susceptibility to Vaginal SHIV_SF162P3_ Infection

**DOI:** 10.1371/journal.ppat.1004567

**Published:** 2014-12-18

**Authors:** Diana Goode, Rosaline Truong, Guillermo Villegas, Giulia Calenda, Natalia Guerra-Perez, Michael Piatak, Jeffrey D. Lifson, James Blanchard, Agegnehu Gettie, Melissa Robbiani, Elena Martinelli

**Affiliations:** 1 Center for Biomedical Research, Population Council, New York, New York, United States of America; 2 AIDS and Cancer Virus Program, Frederick National Laboratory, Frederick, Maryland, United States of America; 3 Tulane National Primate Research Center, Tulane University Sciences Center, Covington, Louisiana, United States of America; 4 Aaron Diamond AIDS Research Center, Rockefeller University, New York, New York, United States of America; Emory University, United States of America

## Abstract

The availability of highly susceptible HIV target cells that can rapidly reach the mucosal lymphoid tissues may increase the chances of an otherwise rare transmission event to occur. Expression of α_4_β_7_ is required for trafficking of immune cells to gut inductive sites where HIV can expand and it is expressed at high level on cells particularly susceptible to HIV infection. We hypothesized that HSV-2 modulates the expression of α_4_β_7_ and other homing receptors in the vaginal tissue and that this correlates with the increased risk of HIV acquisition in HSV-2 positive individuals. To test this hypothesis we used an in vivo rhesus macaque (RM) model of HSV-2 vaginal infection and a new ex vivo model of macaque vaginal explants. In vivo we found that HSV-2 latently infected RMs appeared to be more susceptible to vaginal SHIV_SF162P3_ infection, had higher frequency of α_4_β_7_^high^ CD4^+^ T cells in the vaginal tissue and higher expression of α_4_β_7_ and CD11c on vaginal DCs. Similarly, ex vivo HSV-2 infection increased the susceptibility of the vaginal tissue to SHIV_SF162P3_. HSV-2 infection increased the frequencies of α_4_β_7_^high^ CD4^+^ T cells and this directly correlated with HSV-2 replication. A higher amount of inflammatory cytokines in vaginal fluids of the HSV-2 infected animals was similar to those found in the supernatants of the infected explants. Remarkably, the HSV-2-driven increase in the frequency of α_4_β_7_^high^ CD4^+^ T cells directly correlated with SHIV replication in the HSV-2 infected tissues. Our results suggest that the HSV-2-driven increase in availability of CD4^+^ T cells and DCs that express high levels of α_4_β_7_ is associated with the increase in susceptibility to SHIV due to HSV-2. This may persists in absence of HSV-2 shedding. Hence, higher availability of α_4_β_7_ positive HIV target cells in the vaginal tissue may constitute a risk factor for HIV transmission.

## Author Summary

Understanding the factors that correlate with an increased risk of acquiring HIV infection is key to identify new means of preventing HIV transmission. HSV-2 infection increases the risk of HIV transmission even in absence of visible lesions and inflammation. In order to explore HSV-2− associated factors that could explain this phenomenon, we used a model of asymptomatic HSV-2 infection in macaques and ex vivo cultures of biopsied vaginal tissue. We determined that HSV-2 infection is associated with an increase in subsets of immune cells that express high levels of α_4_β_7_, a molecule needed by the cells to reach the gut and the gut lymphoid tissues. The gut is an important site for HIV infection and pathogenesis and CD4^+^ T cells expressing high levels of α_4_β_7_ (α_4_β_7_^high^) are highly susceptible to the virus. We determined that the HSV-2-driven increase in these cells correlates with an increased susceptibility of the vaginal mucosa to SIV infection. Thus, our results suggest that an increased availability of α_4_β_7_^high^ cells at the mucosal site of HIV exposure may constitute a risk factor for HIV acquisition in HSV-2 positive and, possibly, negative individuals.

## Introduction

Vaginal HIV transmission is a relatively rare event [1] and virions and host characteristics influence the probability of this rare event to occur. Host-related factors include the epithelial and mucus thickness, hormonal environment, presence of inflammation and infection with other sexually transmitted pathogens [2]–[7]. In particular Herpes Simplex Virus Type 2 (HSV-2) infection is associated with a three-fold increased risk of HIV acquisition even in the absence of HSV-2 replication [8], [9].

Clarifying the mechanisms involved in the increased susceptibility of HSV-2 positive individuals to HIV infection may help understanding the characteristics of mucosal microenvironment that facilitate HIV transmission. It was reported that the vaginal mucosa of HSV-2 infected women retains an increased number of CCR5^+^ CD4^+^ T cells long after HSV-2 replication abates. Likewise, plasmacytoid and myeloid dendritic cells (DCs), which infiltrate areas of skin infected with HSV-2, persist after lesion healing even in the context of acyclovir therapy [10], [11]. More recently, an increased total number of CD4^+^ T cells expressing CCR5 and chronic activation markers CD38 and HLA-DR was found in the cytobrush samples of HSV-2 positive asymptomatic women [12]. These factors may partially explain the enhanced risk of HIV acquisition in HSV-2 positive individuals. However, a direct association between the HSV-2-driven increased frequency of these cell subsets and the HSV-2-driven increase in the risk of HIV acquisition has never been demonstrated.

Immune cell trafficking can affect the susceptibility of the genital mucosa to HIV infection by influencing the availability of HIV cell targets at the site of exposure, the immune response to the virus and the ability of infected cells to reach sites of viral expansion and dissemination, such as draining lymph nodes, gut and the gut inductive sites. Thus changes in the expression of integrins or other adhesion molecules influence the susceptibility to vaginal HIV infection. Integrin α_4_β_7_ (α_4_β_7_) is an adhesion molecule specifically involved in trafficking of immune cells in the gut and gut inductive sites [13], [14]. However, α_4_β_7_^+^ cells are also involved in immune response in the vaginal tissue [15]–[17]. CD4^+^ T cells that express high levels of α_4_β_7_, α_4_β_7_^high^ CD4^+^ T cells, are highly susceptible to HIV infection [18]–[20], they are preferentially depleted during acute SIV infection [21] and we recently reported that their frequency correlates with susceptibility to rectal SIV infection [22]. Moreover, administration of a monoclonal antibody (mAb) against α_4_β_7_ prior to intravenous challenge with SIV_mac251_ resulted in lower plasma and tissue viral load and lower proviral DNA compared to the control animals [23]. Notably the animals treated with the anti-α_4_β_7_ mAb showed no signs of progression to AIDS. Finally, pre-treatment with the mAb significantly reduced vaginal SIV infection of rhesus macaques (RM) (Byrareddy et al., Nature, in press).

We previously showed that rectal HSV-2 infection increases the frequency of α_4_β_7_^high^ CD4^+^ T cells at the site of exposure, in the rectal draining LNs and in blood [22]. In this report we explored the possibility that HSV-2 infection increases the frequencies of α_4_β_7_^+^ cell subsets in the vaginal tissue. We further asked if these increases persist in absence of HSV-2 replication in vivo and whether these and other changes in the expression of adhesion molecules correlate with the HSV-2-driven increase in SIV acquisition.

Using a model of HSV-2 vaginally infected macaques, we found that, as in humans, HSV-2 positive animals appeared to be more susceptible to SHIV_SF162P3_ vaginal infection even when challenged with SHIV_SF162P3_ over a year after HSV-2 infection. Notably, HSV-2 positive animals had a trend toward higher frequency of α_4_β_7_^high^ CD4^+^ T cells and higher expression of α_4_β_7_ and CD11c on DCs in the vaginal tissue. Moreover, we found that HSV-2 infection of vaginal tissue ex vivo increased the susceptibility of the tissue to SHIV_SF162P3_ and that specific HSV-2-driven changes in the expression of α_4_β_7_ and other adhesion molecules correlated with the HSV-2-driven increase in susceptibility to SHIV.

## Results

### HSV-2 infected, asymptomatic RMs appear to be more susceptible to vaginal SHIV_SF162P3_ infection

**Figure 1:**
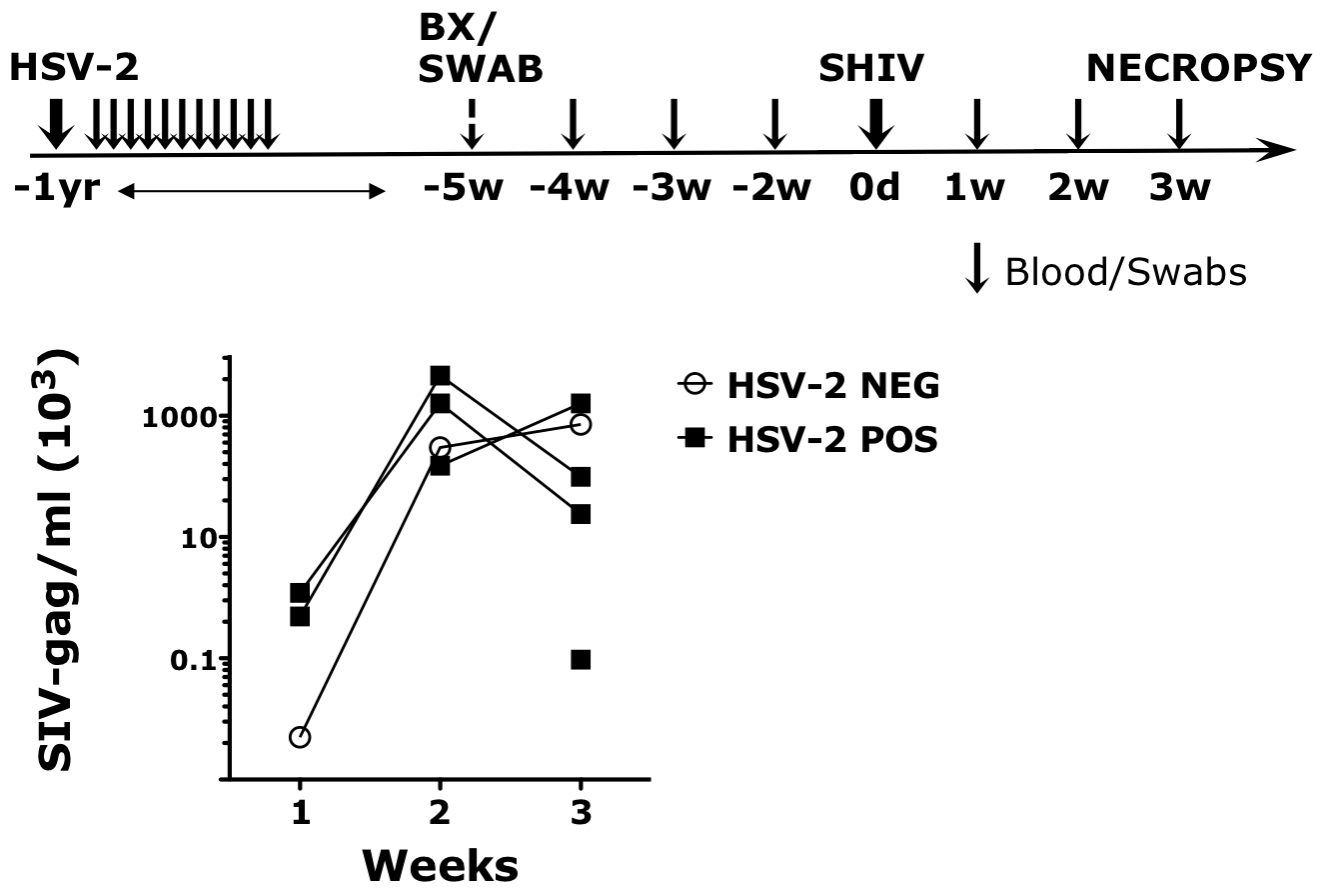
HSV-2 latently infected RMs appear more susceptible to SHIV infection. The upper schematic shows the sampling/challenges schedule of the study. Black arrows represent time point in which blood and swabs were collected. Swabs were never collected at the time of challenge. SHIV_SF162P3_ plasma VLs are shown in the lower graph for the HSV-2 positive (POS) and negative (NEG) RMs for three weeks post challenge. SHIV was detected in the plasma of 4 out of 5 HSV-2 positive RMs.

In humans the risk of HIV acquisition is higher in HSV-2 infected individuals, including asymptomatic subjects [8]. To establish if our HSV-2 macaque model recapitulated the HSV-2/HIV interplay in humans, we compared the susceptibility to SHIV vaginal infection of 5 HSV-2 positive (HSV-2+) and 5 negative (HSV-2−) RMs. The 5 HSV-2+ animals were challenged with HSV-2 one year prior to the start of our study and were confirmed infected by detection of the virus in vaginal swabs at one or more time points after infection (Fig. 1). However, HSV-2 was undetectable at the time when baseline biopsies were taken (5 weeks before SHIV challenge) and 2, 3 and 4 weeks before SHIV challenge (6 out of 6 replicates of HSV-2 nested PCR on vaginal swabs resulted negative). All the animals were challenged vaginally with 250 TCID50 of SHIV_SF162P3_. 4 out of 5 HSV-2+ RMs (80%) acquired SIV, while only 1 out of 5 HSV-2− became SIV+ (20%). The animals were followed for 3 weeks. The 1 HSV-2− SIV+ RM exhibited acute plasma SIV VL similar to the HSV-2+ SIV+ RMs (Fig. 1).

### HSV-2 impacts the expression of integrins in the vaginal tissue

**Figure 2:**
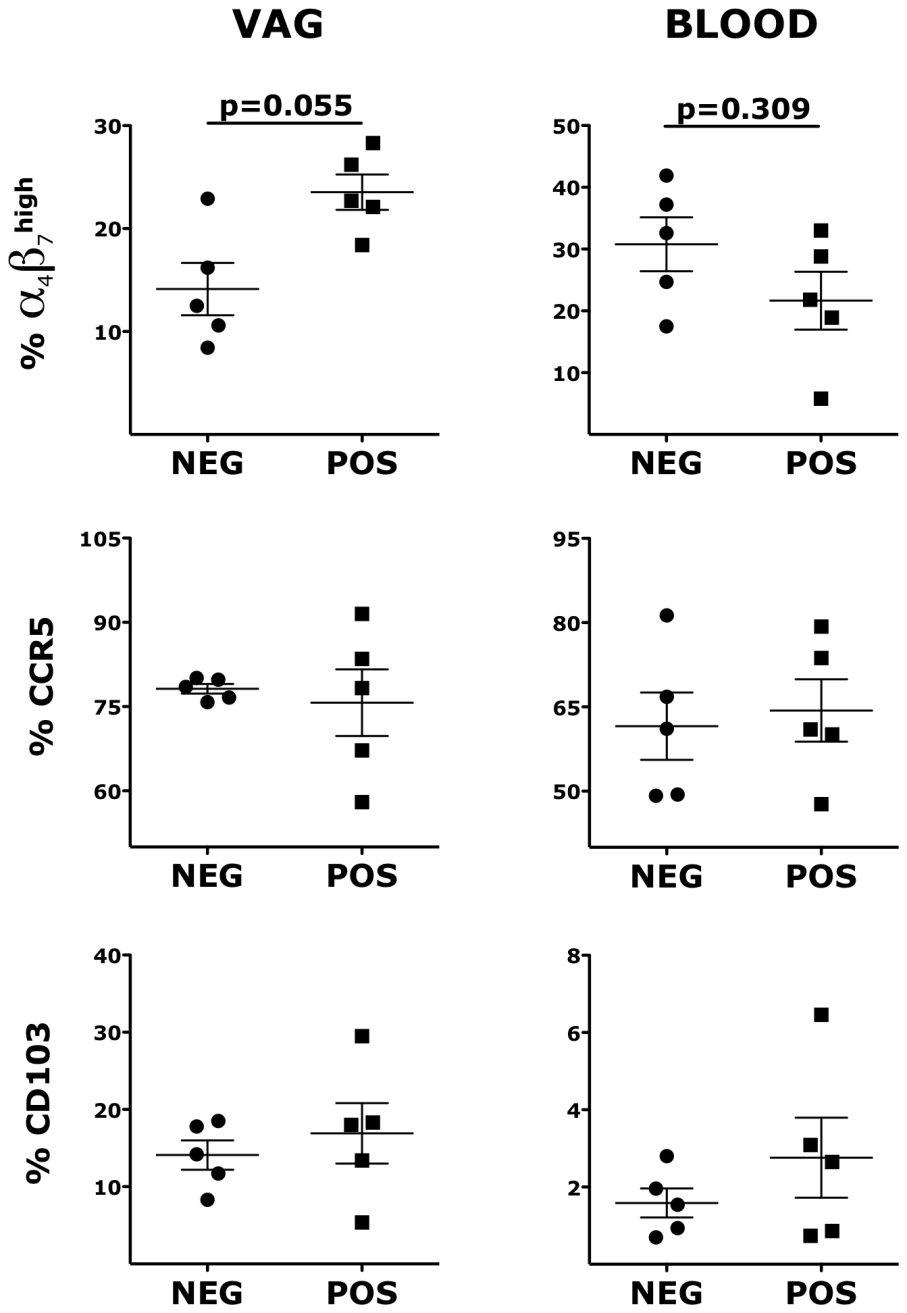
HSV-2 vaginal infection modulates integrin α_4_β_7_ on CD4^+^ T cells. Cells from vaginal tissue and blood were gated on singlets, live, CD3^+^ CD4^+^. The frequencies of different memory subsets are shown for HSV-2 negative (NEG) and HSV-2 positive (POS) RMs. Bars represent mean ± SEM. Unpaired Mann-Whitney test p values are shown when ≤0.125 to indicate a tendency toward a significant difference. p<0.05 was considered significant.

We hypothesized that the increased susceptibility to SHIV_SF162P3_ of the HSV-2 latently infected animals correlated with HSV-2-driven changes in the expression of molecules that are HIV receptors and important for immune cell trafficking in the mucosa. We compared the expression of CCR5, α_4_β_7_ and CD103 on CD4^+^ T cells and CD103, α_4_β_7_, CD11c, CD141 and CD80 on Lin^−^ HLA-DR^+^ DCs in the vaginal tissue and blood of the HSV-2+ RMs with that of the HSV-2− RMs 5 weeks prior to the SHIV challenge. We found that the HSV-2+ RMs had a trend toward higher frequency of α_4_β_7_^high^ memory CD4^+^ T cells (within the CD95^+^; gating strategy depicted in S1 Fig.; p = 0.05) in the vaginal tissue than the HSV-2−, while there was a trend toward a lower frequency in blood (Fig. 2). Notably, the two RMs in the HSV-2+ group that had the highest frequency of α_4_β_7_^high^ CD4^+^ T cells in the vaginal tissue at BL had also the highest peak VL (respectively 46×10^6^ at week 2 and 16×10^6^ at week 3).

In contrast, there was no difference in the frequency of blood and vaginal memory CCR5^+^ CD4^+^ T cells and CD103^+^ CD4^+^ T cells (Fig. 2). We also compared the frequencies of total (not within CD95^+^) α_4_β_7_^high^, CCR5^+^ and CD103^+^ CD4^+^ T cells and again we found a higher frequency of vaginal α_4_β_7_^high^ CD4^+^ T cells in the HSV-2+ RMs (S2 Fig.). No differences were noted for other α_4_β_7_^+^ subsets and in the expression of CD95. Moreover, there was no difference between HSV-2+ and HSV-2− RMs in the total frequency of CD3^+^ and CD3^+^ CD4^+^ T cells in the vaginal tissue (p∼1).

**Figure 3:**
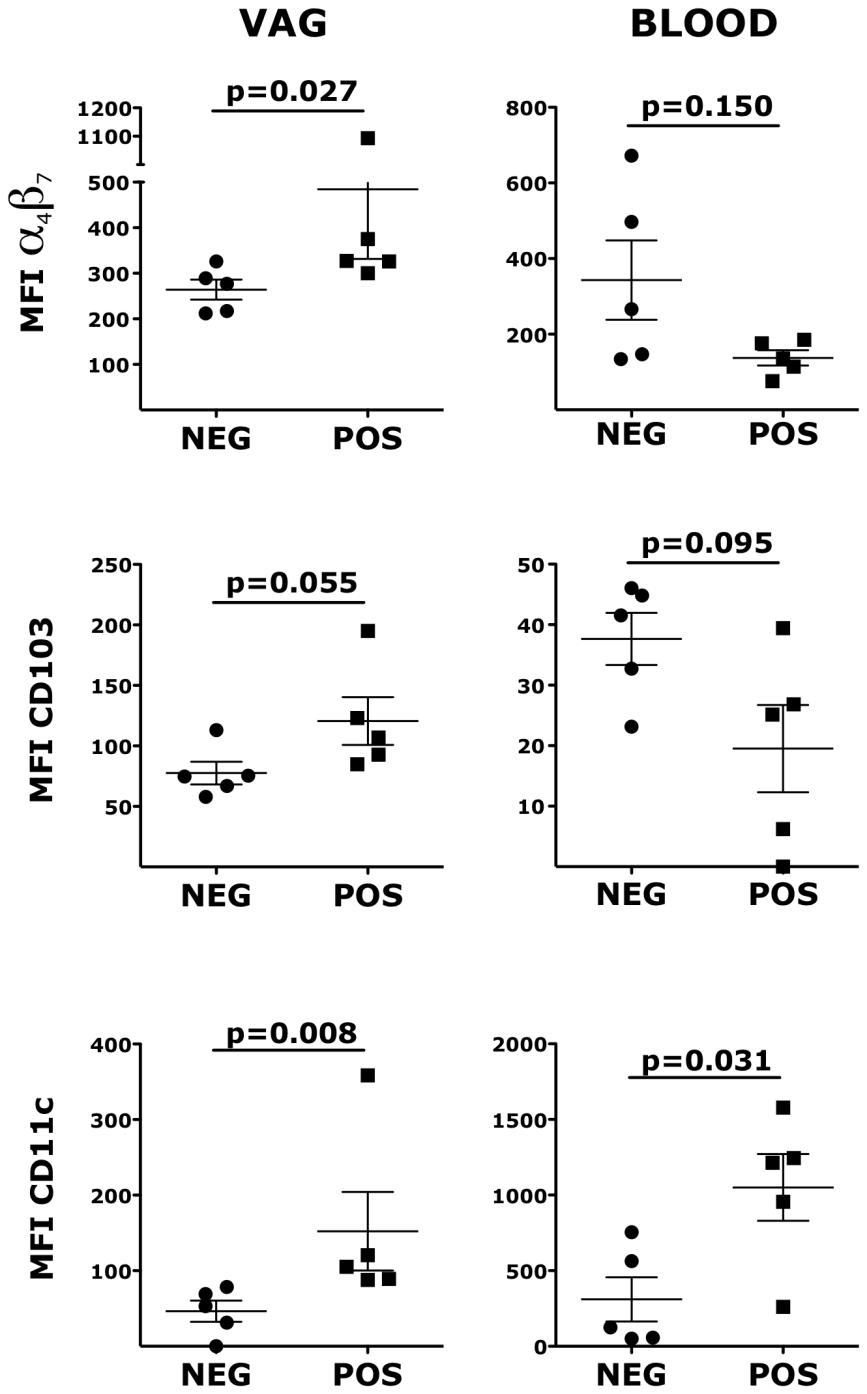
HSV-2 infection impacts the expression of α_4_β_7_, CD103 and CD11c on vaginal DCs. Cells from vaginal tissue and blood were gated on live, singlets and lin^−^ HLA-DR^+^. The frequencies of different subsets are shown for HSV-2 positive and negative RMs. Bars represent mean±SEM. Unpaired Mann-Whitney test p values are shown when ≤0.125 to indicate a tendency toward a significant difference. p<0.05 was considered significant.

Similarly to CD4^+^ T cells, the expression of α_4_β_7_ on vaginal Lin^−^ HLA-DR^+^ DCs was significantly higher in the HSV-2+ RMs compared with the HSV-2−, while in blood there was a tendency toward lower expression (Fig. 3). HSV-2+ vaginal DCs had a trend toward higher expression of CD103 (integrin αE; p = 0.055) and significantly higher expression of CD11c (integrin αX) both in blood and in the vaginal tissues (Fig. 3). Moreover, we found that the HSV-2+ RMs had a trend toward higher frequency of CD141^+^ DCs both in blood (p = 0.055) and in the vaginal tissue (p = 0.093). There was no difference in the expression of HLA-DR and CD80. Finally, we examined blood and vaginal pDCs, but there was no difference in the expression of α_4_β_7_, CD103, CD141 and CD80.

Previously, we described a strong positive correlation between the frequencies of α_4_β_7_^high^ CD4^+^ T cells in blood and rectal mucosa [22]. However, the finding of this study suggested no correlation between blood and vaginal tissue. To verify this, we analyzed data from 25 SIV uninfected animals when blood and vaginal biopsies were collected on the same day. Indeed, we found a trend toward a negative correlation in the frequency of α_4_β_7_^high^ CD4^+^ T cells between blood and vaginal mucosa and this was independent of the HSV-2 status of the RMs (S3 Fig.).

### Inflammatory cytokines and chemokines persist in vaginal fluids of HSV-2 latently infected animals

**Figure 4:**
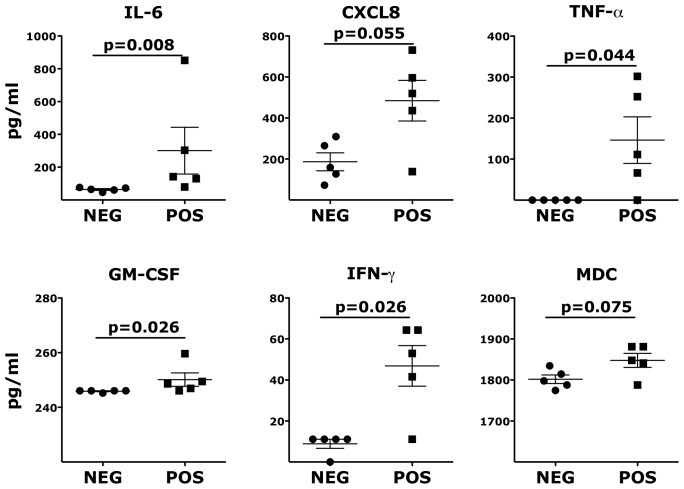
Inflammatory cytokines and chemokines persist in vaginal fluids of HSV-2 latently infected animals. The concentrations of soluble factors in the RMs' vaginal swabs are shown. Bars represent mean ± SEM. Unpaired Mann-Whitney test p values are shown when ≤0.125 to indicate a tendency toward a significant difference. p<0.05 was considered significant.

To further explore factors potentially associated with the increased susceptibility of the HSV-2 latently infected animals, we examined the levels of 29 different soluble proteins in the vaginal fluids of the HSV-2+ and HSV-2− RMs 2 weeks before SHIV challenge. We found that the HSV-2+ animals had significantly higher levels of IL-6, TNF-α, GM-CSF and IFNγ compared to the HSV-2− animals (Fig. 4). There was also a trend for a higher concentration of CXCL8 and the macrophage-derived chemokine (MDC) (Fig. 4) and for IL-1β, CCL3 and IL-12p70 (S4 Fig.).

### Ex vivo HSV-2 infection increases the susceptibility of the vaginal mucosa to SHIV_SF162P3_

**Figure 5:**
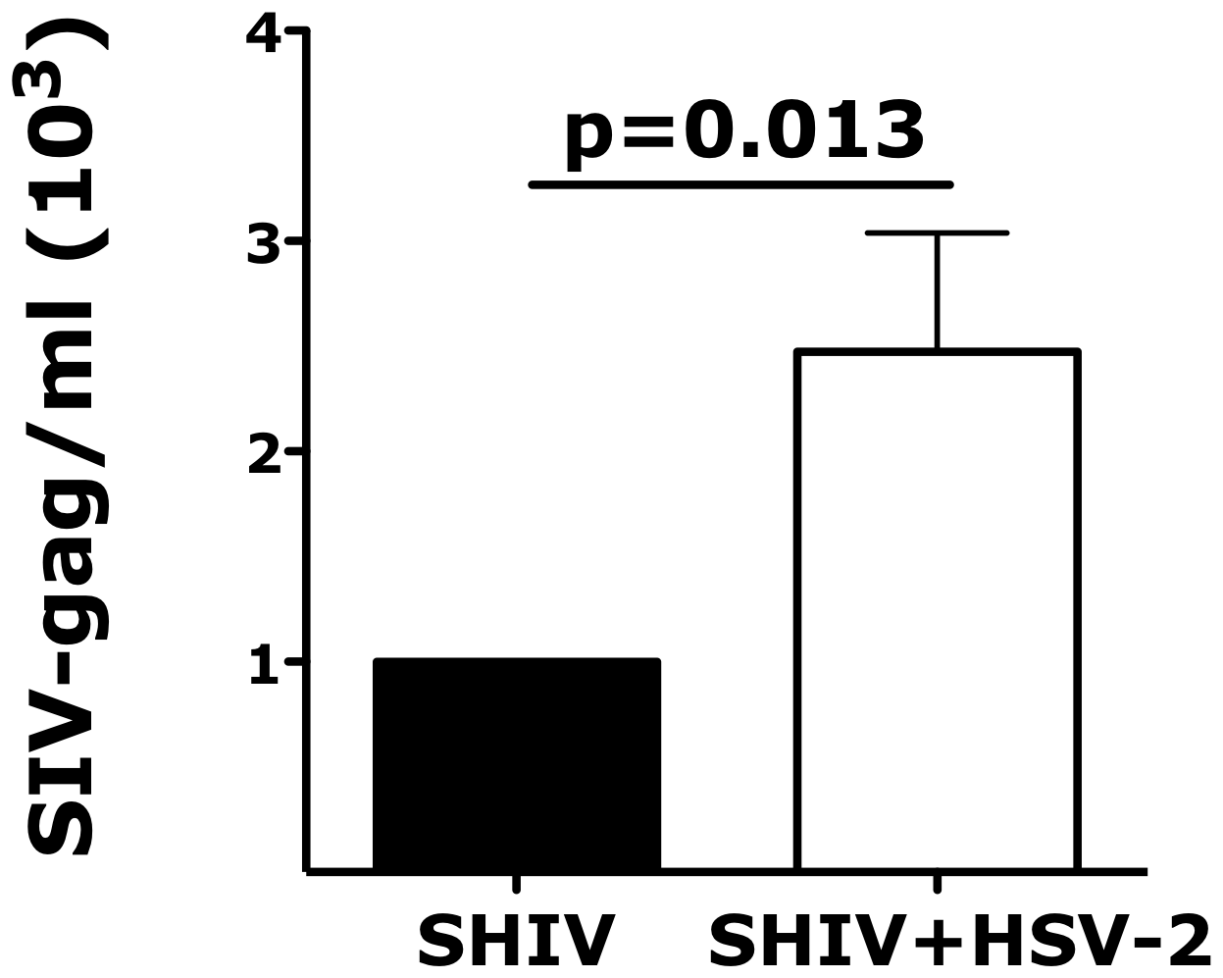
Vaginal tissues infected ex vivo with HSV-2 are more susceptible to SHIV infection. The fold increases in SHIV copies/ml of the vaginal explants co-infected with HSV-2 are shown compared to the SHIV copies/ml of vaginal explants infected with SHIV alone (set as 1). Mean ± SEM are shown from n = 11 independent experiments; each experiment = 1 RM; each condition run in duplicate. Paired Wilcoxon signed-rank test p values are shown. p<0.05 was considered significant.

To further evaluate the impact of HSV-2 infection on the vaginal mucosa and how this modulates the susceptibility to SHIV_SF162P3_, we developed a novel ex vivo model of HSV-2 and SHIV infection of RM vaginal tissue polarized cultures. This model, a modified version of the model described in Cummins et al. [24], [25], allowed us to investigate the early events after HSV-2 infection and HSV-2/SHIV co-infection and to distinguish the effect of HSV-2 on cells migrating outside the mucosa from cells retained in the mucosa. In this system, RM vaginal biopsies, equalized in size with 3 mm skin biopsies punches, were placed on a transwell insert inside a hole with the mucosa facing up and matrigel was applied to seal the hole [25]. This resulted in a neat separation of the mucosal tissue from the cells migrating out of the tissue. The vaginal tissue was infected with HSV-2 in presence (uninfected control) or absence of acyclovir (ACV) for 3 hours. Then the tissues were washed and infected with SHIV_SF162P3_ for 18 hours. The infected tissues were washed and cultured for additional 3 days. HSV-2 infected and uninfected tissues were cultured in parallel with tissues infected with SHIV alone or infected with the two viruses. In agreement with our in vivo results, the HSV-2 infected vaginal explants were significantly more susceptible to SHIV infection than the HSV-2 uninfected (Fig. 5).

### Ex vivo HSV-2 infection impacts the expression of α_4_β_7_ and the release of inflammatory cytokines

**Figure 6:**
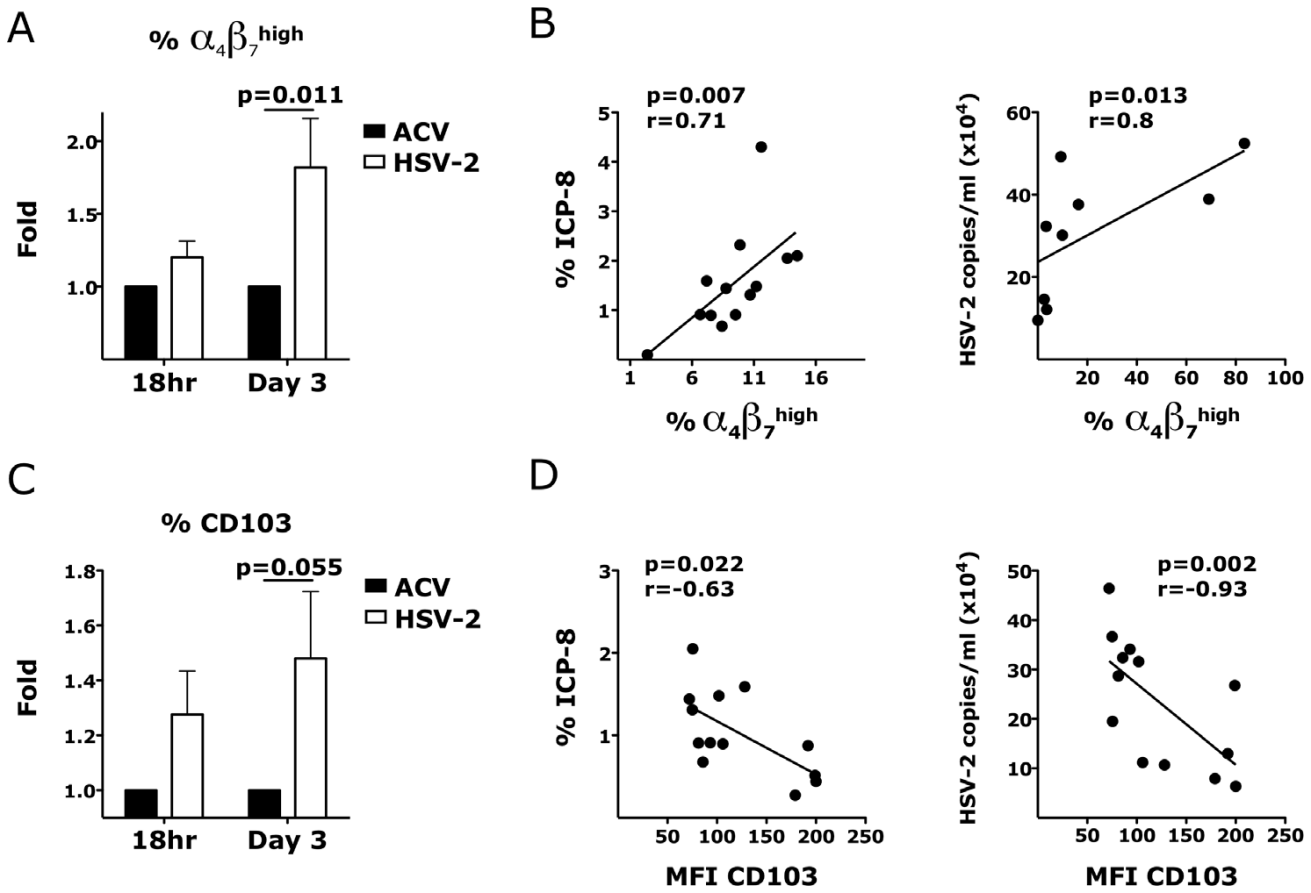
HSV-2 ex vivo infection impacts the expression of integrins in the vaginal mucosa. A and C) The fold increases in the frequency of α_4_β_7_^high^ CD4^+^ T cells (A) and MFI of CD103^+^ CD4^+^ T cells (C) in HSV-2 infected vaginal tissues (HSV-2) are shown compared to the tissues treated with HSV-2 in presence of Acyclovir (uninfected control; ACV; set as 1) 18 hours and 3 days after HSV-2 infection (mean ± SEM, n = 21 independent experiments; each experiment = 1 RM; each condition run in duplicate). Paired Wilcoxon signed-rank test p values are shown. p<0.05 was considered significant. (B and D) The correlation between the frequency of α_4_β_7_^high^ CD4^+^ T cells (B) and MFI of CD103^+^ CD4^+^ T cells (D) with the frequency of HSV-2^+^ cells (ICP-8^+^) (left) and HSV-2 copies/ml (right) are shown (each dot represents 1 explant; n = 8–13). Fitting linear regression lines and Spearman rank correlations p values are shown. p<0.05 was considered significant.

Consistent with the in vivo data, the HSV-2 infected vaginal tissues had higher frequency of α_4_β_7_^high^ memory CD4^+^ T cells than the control tissues 3 days after infection. A tendency toward a higher frequency of α_4_β_7_^high^ CD4^+^ T cells was found also after 18 hours (p = 0.181; Fig. 6A). Notably, the frequency of α_4_β_7_^high^ CD4^+^ T cells in the vaginal mucosa 3 days after HSV-2 infection correlated with HSV-2 replication (Fig. 6B). Moreover, we found that CD4^+^ T cells in the mucosa of HSV-2 infected explants had a significantly higher expression of CD103 3 days after HSV-2 infection than the uninfected tissues (Fig. 6C) and a non-significantly higher expression after 18 hours (p = 0.185; Fig. 6C). The frequency of CD103^+^ CD4^+^ T cells was also increased after 18 hours (p = 0.112) and 3 days (p = 0.055). However, the expression of CD103 inversely correlated with HSV-2 replication. This may be explained by a decrease in the expression of CD103 with an increase in cell death due to HSV-2 replication (S5 Fig.). We found no difference in the frequencies of CCR5^+^, CCR6^+^, CD62L^+^ and CCR7^+^ CD4^+^ T cells or in the expression of these markers between HSV-2 infected and control tissues.

**Figure 7:**
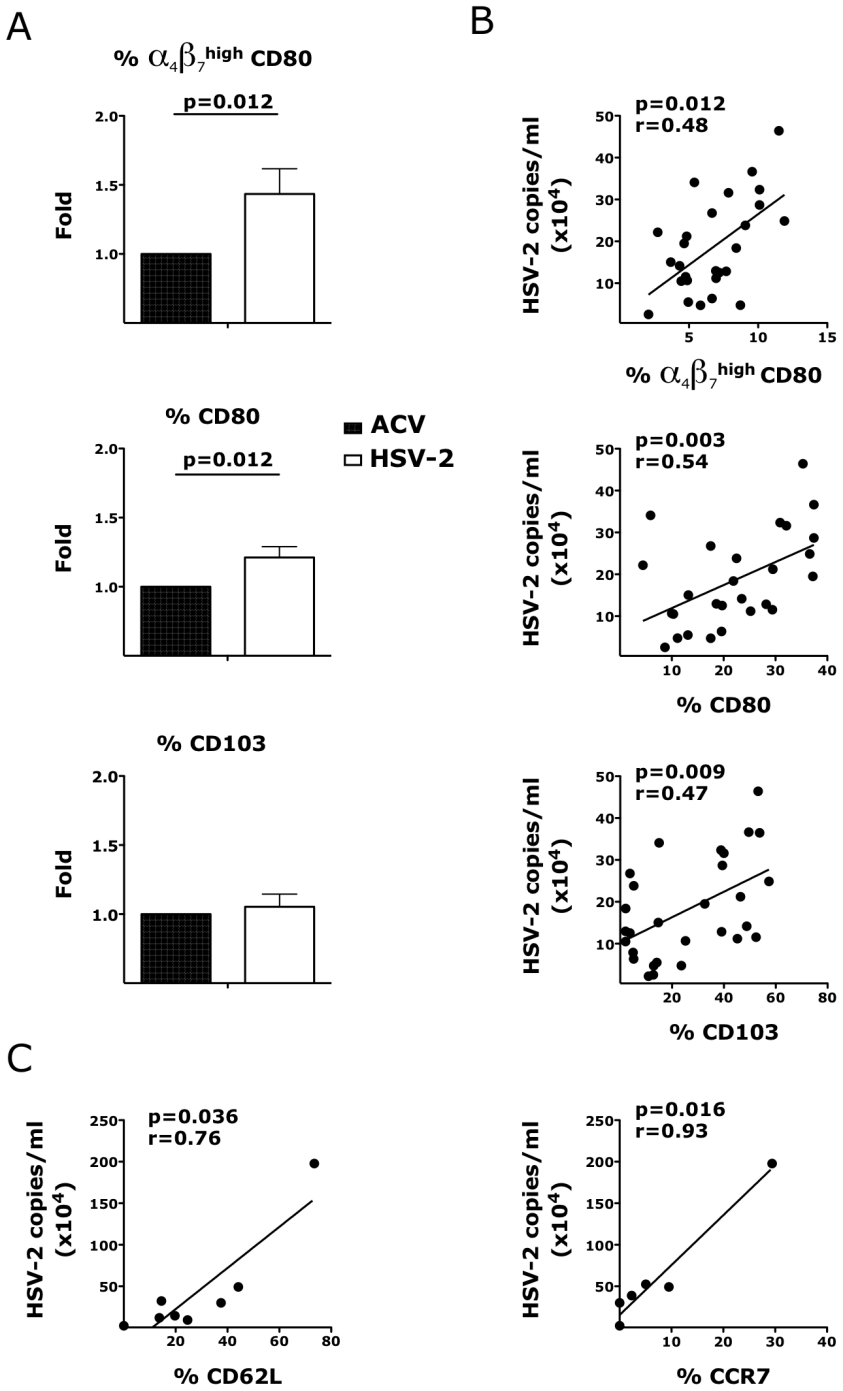
HSV-2 impacts the expression of integrins and co-stimulatory molecules on CD3^−^ HLA-DR^+^ cells. Cells were gated on singlets, live, CD3^−^ HLA-DR^+^. A) The fold increases in the frequencies of α_4_β_7_^high^ CD80^+^, CD80^+^ and CD103^+^ DCs in HSV-2 infected vaginal tissues are shown compared to the tissues treated with HSV-2 in presence of Acyclovir (uninfected control; ACV; set as 1) 3 days after HSV-2 infection. Means ± SEM are shown from n = 21 independent experiments; each experiment = 1 RM; each condition run in duplicate. Paired Wilcoxon signed rank p values are shown (p<0.05 is considered significant). B–C) The frequencies of α_4_β_7_^high^ CD80^+^, CD80^+^, CD103^+^, CD62L^+^ and CCR7^+^ DCs in the mucosa at day 3 are plotted against the HSV-2 copies/ml (each dot represents 1 explant; n = 27–29). Fitting linear regression lines, Spearman rank correlation p values and correlation coefficients r are shown. p<0.05 was considered significant.

We then investigated if ex vivo HSV-2 infection would also modulate the expression of integrins on CD3^−^ HLA-DR^+^ antigen presenting cells (APCs) in the vaginal mucosa. Although, there was no HSV-2-driven increase in the expression of α_4_β_7_ on all the APCs in the tissue, we found that the frequency of α_4_β_7_^high^ CD80^+^ APCs 3 days after infection was higher in the HSV-2 infected tissues than in the controls (Fig. 7A). We examined this population because in an earlier study we found that a small population of α_4_β_7_^high^ CD80^+^ DCs correlated with SIV rectal infection [22]. The frequency of CD80^+^ APCs was also significantly higher and the frequencies of both subsets correlated with HSV-2 replication (Fig. 7B). A non-significant increase in the α_4_β_7_^high^ cells within the CD80^+^ APCs (p = 0.125) suggests that the changes in α_4_β_7_ are independent of the increase in CD80. Interestingly, although there was no difference in the frequencies of CD103^+^, CD62L^+^, CCR7^+^ APCs in the HSV-2 infected vaginal mucosa (Fig. 7A), the frequencies of these subsets directly correlated with HSV-2 replication (Fig. 7B and 7C).

**Figure 8:**
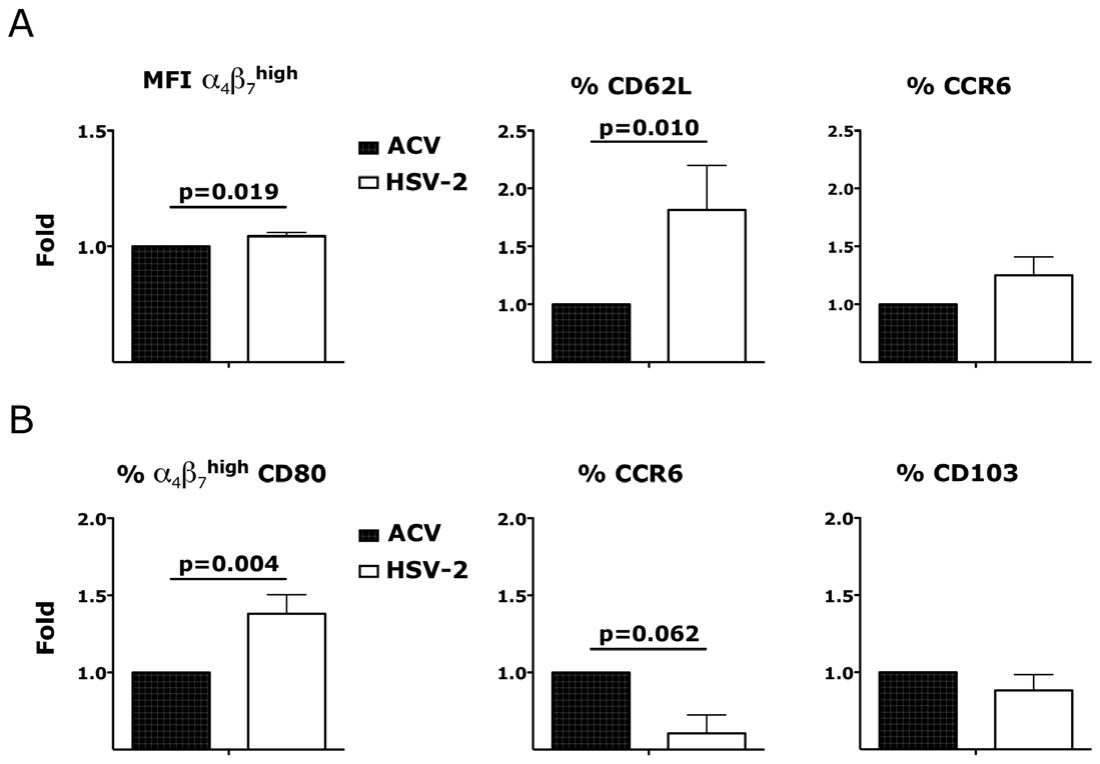
HSV-2 modulates the phenotype of migratory CD4^+^ T cells and CD3^−^ HLA-DR^+^ cells. A) The fold increases in the MFI of the α_4_β_7_^high^ CD4^+^ T cells (left) and the frequencies of CD62L^+^ (center) and CCR6^+^ (right) CD4^+^ T cells that migrated out of the HSV-2 infected mucosa 18 hours after infection are shown compared to the Acyclovir control (+ACV, set as 1; n = 13–19). B) The fold increases in the frequencies of α_4_β_7_^high^ CD80^+^ (left) CCR6^+^ (center) and CD103^+^ (right) CD3^−^ HLA-DR^+^ cells that migrated out of the HSV-2 infected mucosa 3 days after infection are shown compared to the Acyclovir control (+ACV, set as 1; n = 6–13). Bars represent mean ± SEM. Wilcoxon signed-rank test p values are shown. p<0.05 was considered significant.

Since the rate of HIV systemic dissemination could be greatly influenced by the ability of infected cells to leave the tissue and traffic to the GALT, we explored the effect of HSV-2 on the first cells that migrated out of the vaginal tissue ex vivo. We found that the migratory CD4^+^ T cells in the HSV-2 infected explants had higher α_4_β_7_ expression in the α_4_β_7_^high^ subset 18 hours after HSV-2 infection compared to the control tissues (Fig. 8A). The frequency of migratory CD62L^+^ CD4^+^ T cells was also significantly higher in the HSV-2 infected conditions than in the uninfected. In contrast, there was no difference in the frequency of migratory CCR6^+^ CD4^+^ T cells (Fig. 8A; p = 0.232) or in the migratory CCR5^+^, CD62L^+^, CCR7^+^ CD4^+^ T cells (p∼1). Finally, there was a significant increase in the migratory α_4_β_7_^high^ CD80^+^ APCs in the HSV-2 infected tissues compared to the uninfected controls and a non-significant decrease in migratory CCR6^+^ APCs (Fig. 8B) 3 days after HSV-2 infection. No significant change in migratory CD103^+^ or CCR7^+^ APCs was noted (Fig. 8B).

**Figure 9:**
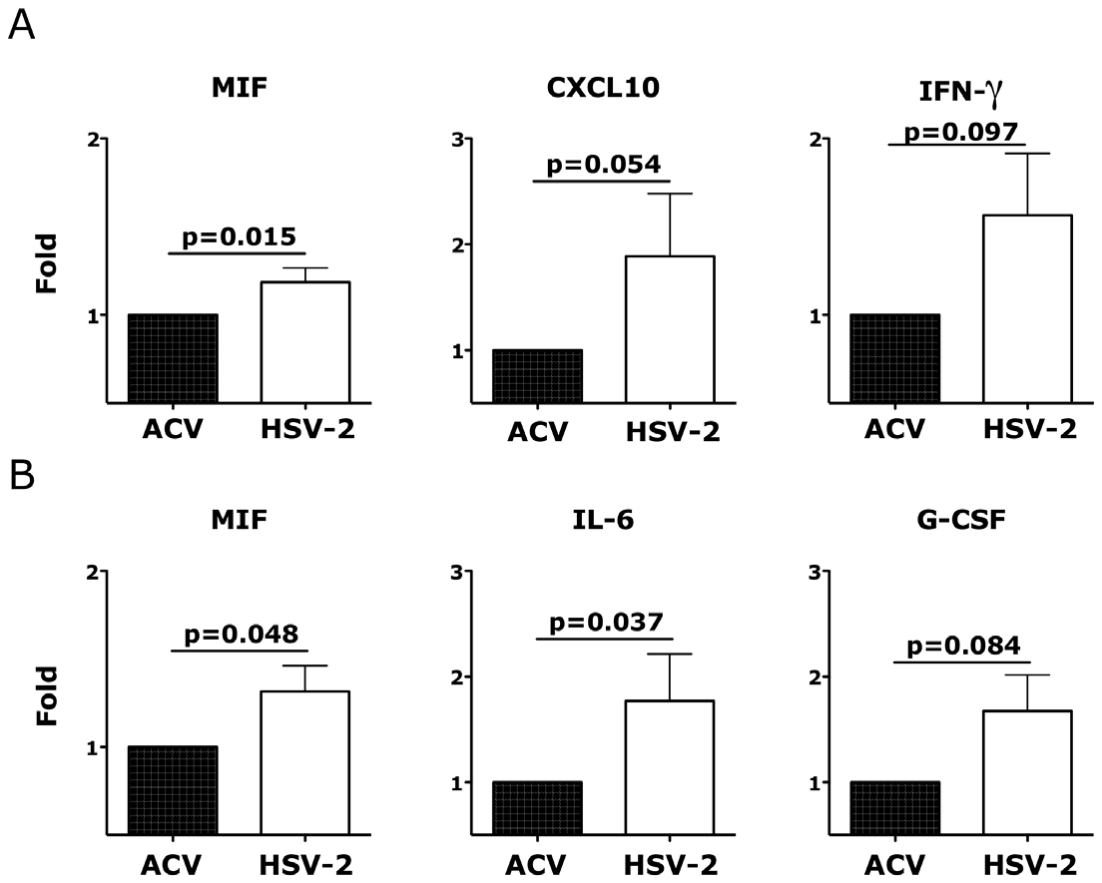
Ex vivo HSV-2 infection modulates the release of soluble factors by vaginal mucosa. The fold increases in the concentrations (pg/ml) of the indicated factors are shown from the supernatant 18 hours (A) and 3 days (B) after HSV-2 infection compared to Acyclovir control (+ACV, set as 1; n = 5–10). Bars represent mean±SEM. Wilcoxon signed-rank test p values are shown when ≤0.125 to indicate a tendency toward a significant difference. p<0.05 was considered significant.

Exploring the effect of HSV-2 infection on the release of soluble factors, we found that, after 18 hours, HSV-2 infection of vaginal tissue induced the release of the inflammatory macrophage migration inhibitory factor (MIF), CXCL10 (IP-10) and a trend toward an increased release of IFNγ (Fig. 9A) and of the epidermal grow factor (EGF; p = 0.125). After 3 days the concentration of MIF was still higher in the HSV-2 infected tissues compared to the controls, while there was also a significant increase in IL-6 and a almost significant increase in the granulocyte-colony stimulating factor (G-CSF) (Fig. 9B).

### The HSV-2-driven increase in α_4_β_7_ expression correlates with the susceptibility of the vaginal mucosa to SHIV_SF162P3_

**Figure 10:**
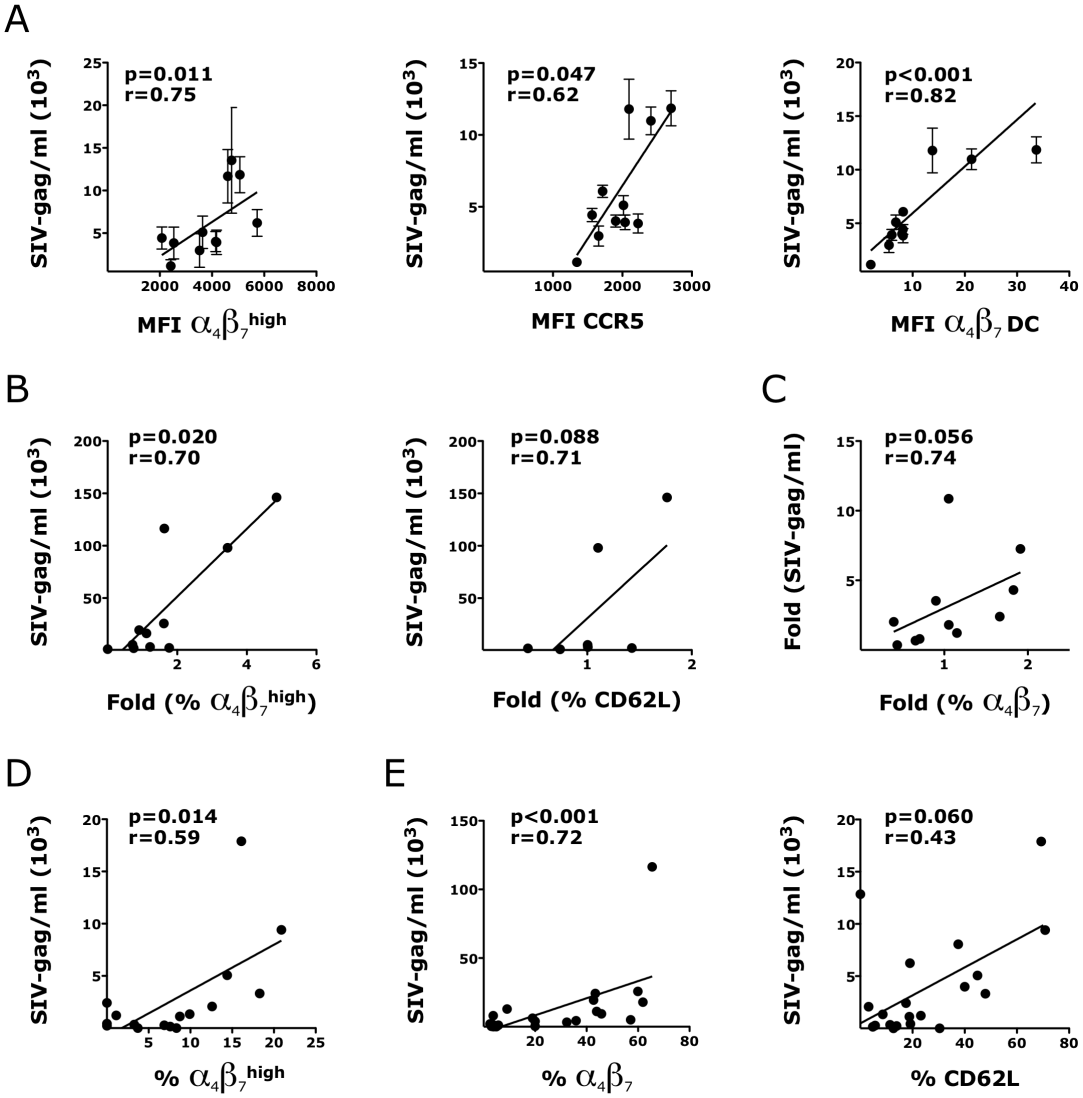
HSV-2-driven changes in T and DCs phenotype in the vaginal mucosa correlate with increased susceptibility to SHIV_SF162P3_. A) The expression of α_4_β_7_^high^ and CCR5^+^ CD4^+^ T cells and the frequency of α_4_β_7_ DCs (Lin^−^ HLA-DR^+^) prior to infection are plotted against SHIV copies/ml in the supernatant of the same explant 3 days after SHIV infection (all SHIV alone; n = 3–12 replicates per RM). B) The fold increases in the frequencies of α_4_β_7_^high^ and CD62L^+^ CD4^+^ T cells in the HSV-2 infected explants (HSV-2 alone; no SHIV) compared to the Acyclovir control (uninfected) from the same RM are plotted against the SHIV copies/ml in the sups of SHIV/HSV-2 co-infected explants from the same animal 3 days after co-infection (n = 7–11 RMs; duplicates/condition from each animal were averaged before calculating the fold increases). C) The correlation between the fold increases in the frequency of α_4_β_7_^+^ CD3^+^ HLA-DR^−^ cells in the HSV-2 infected explants (HSV-2 alone; no SHIV) compared to the Acyclovir control (uninfected; set as 1) are plotted against the fold increases in the SHIV copies/ml in the supernatants of the HSV-2/SHIV co-infected explants compared to the SHIV alone condition from the same animal (set as 1) 3 days after HSV-2/SHIV infection (n = 11 RMs; duplicates/condition from each animal were averaged before calculating the fold increases). D–E) The frequencies of migratory α_4_β_7_^high^ CD4^+^ T cells (D) and mucosal α_4_β_7_^+^ and CD62L^+^ DCs (E) in each SHIV infected explants are plotted against the SHIV copies/ml in the supernatant of the same explants 3 days after SHIV infection (all the SHIV alone and SHIV/HSV-2 explants were included; n = 16–23). In some of the experiments CD62L was not added to the panel and/or the staining of migratory cells was not performed. Fitting linear regression lines, Spearman rank correlation p values and correlation coefficients r are shown. p<0.05 was considered significant.

In a set of experiments performed using a simple immersion model of SHIV_SF162P3_ infection of vaginal explants, we found that, in the absence of HSV-2, the expression of α_4_β_7_ and CCR5 on CD4^+^ T cells and the frequency of α_4_β_7_^+^ HLA-DR^+^ Lin^−^ DCs at base line (prior to infection) correlated with SHIV_SF162P3_ replication 3 days post-infection (Fig. 10A). Thus, we investigated in our new model of vaginal explants, whether the HSV-2-driven increase in the frequency of α_4_β_7_^high^ CD4^+^ T cells could be correlated with the HSV-2-driven increase in SHIV infection. Confirming our hypothesis, we found that the higher was the HSV-2-driven increase in the frequency of α_4_β_7_^high^ CD4^+^ T cells (in the HSV-2 infected tissues compared with the control HSV-2− tissues) the higher was the SHIV replication in the HSV-2/SHIV co-infected tissues. Specifically, the fold increase in the frequency of α_4_β_7_^high^ CD4^+^ T cells in the HSV-2+ SHIV+ tissues compared with the HSV-2− controls directly correlated with the SHIV replication in the HSV-2+SHIV+ tissues, evaluating matched cultures from the same animal (Fig. 10B). A similar trend, although non-significant, was found for the increase in the CD62L^+^ CD4^+^ T cells (Fig. 10B). Moreover, we found that the HSV-2-driven increase in the frequency of α_4_β_7_^+^ APCs (HSV-2 only vs ACV control) trended toward a direct correlation with the HSV-2-driven increase in SHIV infection (HSV-2/SHIV co-infection compared to the SHIV only condition in matched cultures from the same animal; Fig. 10C). No other HSV-2-induced change or frequency of CD4^+^ T cells subsets (CCR5^+^, CCR6^+^, CD103^+^ and CCR7^+^) and APCs subsets (CD103^+^, CD62L^+^, CCR7^+^ and CD141^+^) examined could be correlated with SHIV replication or with an increase in SHIV replication (in HSV-2/SHIV co-infected vs SHIV alone; p values >0.2).

Interestingly, within the same SHIV infected tissue, independent of the presence of HSV-2, the frequencies of migratory α_4_β_7_^high^ CD4^+^ T cells (Fig. 10D) and α_4_β_7_^+^ HLA-DR^+^ cells within the mucosa (Fig. 10E, left) strongly correlated with SHIV replication 3 days after infection. A similar tendency was found for mucosal CD62L^+^ APCs (Fig. 10E, right).

As expected, the vaginal tissues infected with SHIV alone released significantly more pro-inflammatory factors compared to the uninfected (ACV control; S1 Table). However, interestingly, when HSV-2 was present (SHIV+HSV-2 condition) some of the cytokines that were released in a significantly higher amount by the explants infected with SHIV alone, were no longer significant (S1 Table). This suggests that HSV-2 may dampen the inflammatory response to SHIV. In fact, comparing the cytokine profile of SHIV with that of HSV-2, there were numerous significant differences (Table S2).

## Discussion

Several groups have shown that HSV-2 increases the frequency of HIV target cells at the site of HSV-2 infection and that these cells persist in the mucosa in absence of HSV-2 shedding [10], [17], [26]. However, a direct correlation between the HSV-2-driven increase in the frequency of these cell subsets and the increased risk of HIV and SIV acquisition has never been shown. We used RMs that were infected vaginally with HSV-2 1 year before the start of this study to explore the differences in the microenvironment of the vaginal mucosa and the vaginal fluids between HSV-2 infected, asymptomatic animals and uninfected. We found that, as in humans, even in absence of visible lesions or demonstrable active HSV-2 replication, the HSV-2+ animals appeared to be more susceptible than the HSV-2− to SHIV vaginal infection (although the study is underpowered to detect a significant difference). The presence of very low levels of HSV-2 replication in the tissues, below the sensitivity of our assay, cannot be ruled out. Moreover, since HSV-2 shedding is sporadic, it is possible that we missed it because of the infrequency of sampling. The same uncertainty is reported for human subjects [27]. Susceptibility to HIV and SIV infection may vary with the stage of the menstrual cycle and we have shown that the levels of progesterone and estrogens may impact the frequency of α_4_β_7_^+^ cells in the endocervix [28], [29]. The RMs in the present study were not treated with depot medroxyprogesterone acetate (DMPA) and it is possible that sex hormones played a role in the differential susceptibility to SHIV infection of the HSV-2+ and HSV-2− RMs. However, it is unlikely that the HSV-2+ animals had a synchronized menstrual cycle and that at the time of challenge they were all in the follicular phase, which has been suggested to constitute a “window of vulnerability” to HIV infection [29].

In addition to our in vivo experiments, to thoroughly dissect the effect of HSV-2 on the vaginal mucosa, we used a novel ex vivo model of HSV-2 infection of macaque vaginal tissue. Importantly, this model allowed us to determine which changes in the mucosal microenvironment directly correlated with higher susceptibility to SHIV. As with our in vivo studies, we found that the HSV-2 infected vaginal explants were more susceptible to SHIV infection. However, the ex vivo system modeled the acute phase of HSV-2 infection, and, as such, differed from the in vivo study which modeled the asymptomatic stage. Although, the in vivo study was underpowered and we could not have drawn any conclusion from the in vivo data alone, the comparison of the results obtained in vivo and ex vivo suggests that at least some of the HSV-2-driven changes that we have determined ex vivo persist in vivo after the HSV-2 replication abates. Notably, both in vivo and ex vivo, we found a higher frequency of α_4_β_7_^high^ CD4^+^ T cells in HSV-2 infected vaginal tissue after HSV-2 infection. Although in vivo this did not quite reach significance likely because of the very small number of animals per group, together with the ex vivo findings, our results suggest HSV-2 infection directly increases the availability of α_4_β_7_^high^ CD4^+^ T cells in the vaginal mucosa and this effect appears to persist long after the resolution of the acute phase of HSV-2 infection. This finding is particularly relevant in HIV infection because after exposure to SIV/HIV the α_4_β_7_^high^ CD4^+^ T cells migrate more rapidly to the GALT than α_4_β_7_^low^ or α_4_β_7_^−^. A higher rate of infection of the gut is likely associated with a more rapid viral dissemination and a more profound damage to the intestinal mucosa. While we focused this work on the impact of HSV-2 on SHIV acquisition and the factors associated with it, further analysis of the tissues of the HSV-2/SHIV co-infected animals will help to determine if HSV-2 truly influences the distribution of SHIV in different anatomical sites. This is a planned follow up to the present study.

In contrast to a previous report [10], we found no difference in the vaginal mucosa in the frequency of CCR5^+^ CD4^+^ T cells. The apparent contradiction can be explained by the fact that the frequency of CCR5^+^ CD4^+^ T cells in that report was compared between areas of the genital mucosa where lesions were present and unaffected areas within the same subject. In our macaque model, since we did not detect lesions, the vaginal mucosa was sampled randomly. Thus, we report that the overall frequency of CCR5^+^ CD4^+^ T cells within the entire vaginal tissue of HSV-2 infected macaques is not different than in HSV-2 uninfected animals.

CD103 (integrin α_E_) is expressed on lymphocytes that traffic to the peripheral mucosa and its receptor, E-Cadherin, is expressed mainly on epithelial cells [14], [30]. CD103 pairs with β_7_ to form α_E_β_7_, which mediates T cells adhesion to epithelial cells and can influence the epithelial cells function [31]. While we found no difference in vivo, ex vivo HSV-2 infection of the vaginal mucosa increased the frequency of CD103^+^ CD4^+^ T cells. This may be explained by the active HSV-2 replication ex vivo compared to the latent infection in vivo. However, the increase in the CD103^+^ T cells during HSV-2 acute infection may not persist once the infection abates.

In contrast to the findings in mucosa, there was no significant difference in the frequency of blood α_4_β_7_^high^ CD4^+^ T cells. In blood, the frequency of this population tended to be lower in the HSV-2+ animals than in the negative. This agrees with a trend towards an inverse correlation in the frequency of α_4_β_7_^high^ memory CD4^+^ T cells between blood and vaginal tissue. It is important to note that these data suggest that the correlation of α_4_β_7_^high^ memory CD4^+^ T cells between blood and rectal tissue that we describe previously [22] does not apply to the vaginal tissue. Therefore the association between susceptibility to rectal infection and the frequency of α_4_β_7_^high^ memory CD4^+^ T cells in blood does not occur in the context of vaginal SIV infection.

Our results also differ from those described by another report [12]. Shannon et al. found that women with asymptomatic HSV-2 infection had a higher frequency of blood α_4_β_7_^+^ CD4^+^ T cells. We found no difference in the frequency of the entire population of α_4_β_7_^+^ CD4^+^ T cells in blood. The discrepancy may be due to a difference in the method used to detect the α_4_β_7_ population. While in our work we used a clone against the dimeric form of the integrin, in Shannon et al. the population was identified by the double staining of α_4_ and β_7_. Both molecules can form dimers with other α and β chains and be present on the same cells as α_4_β_1_ and α_E_β_7_. Thus, the frequency of cells that co-express α_4_ and β_7_ may differ from that of cells that express the α_4_β_7_ dimer (our population of interest).

DCs that reside in submucosal tissues have been proposed as among the first cells that encounter HIV following sexual transmission [32], [33]. Herein we report that vaginal DCs in HSV-2+ animals express higher level of α_4_β_7_ than in HSV-2− RMs. Thus, after mucosal HIV exposure, the HIV loaded DCs in the vaginal tissue of HSV-2+ individuals may travel more efficiently to the GALT than vaginal DCs in HSV-2−, causing a more rapid viral spread. On the other hand, ex vivo acute HSV-2 replication significantly increased the frequency of α_4_β_7_^high^ CD80^+^ APCs, while no increase in α_4_β_7_ expression was noted. Virtually all tissue APCs have the potential to transfer the virus to the T cells [34]. However, HSV-2 may affect the expression of α_4_β_7_ only on specific DCs subset (e.g. migratory DCs). Vaginal DCs of HSV-2+ RMs expressed also higher levels of CD103 and CD11c. Mucosal CD103^+^ DCs are migratory DCs that populate the lamina propria (LP) especially of the intestinal tract. An increase in CD103 expression by DCs may contribute to HIV transport to the gut LP. CD11c is also an important adhesion molecule. In its dimeric form with CD18 (β_2_ chain), CD11c can bind ICAM-1 [35] and this interaction may increase the formation of cell-cell synapses enhancing HIV replication.

Mucosal chemokines and cytokines released in the vagina influence the local immune environment and modulate cell migration. We found that the vaginal fluids of HSV-2+ RMs contained more pro-inflammatory molecules and Th1 skewing cytokines than fluids from HSV-2− animals. Since the soluble factors were measured at a time when no HSV-2 and no lesions could be detected, we can assume that a pro-inflammatory vaginal microenvironment persisted in HSV-2+ RMs in absence of HSV-2 symptomatic infection. This is particularly relevant since a pro-inflammatory environment could increase the activation status of HIV target cells rendering them more permissible to HIV replication. Interestingly, HSV-2 and SHIV have very different cytokine profiles ex vivo and the presence of HSV-2 seems to decrease the inflammatory response to SHIV.

We also found that HSV-2 infection ex vivo modulates the phenotype of the cells migrating out of the tissue early during the culture. Although after few days the fact that cells leave the tissue ex vivo may be the result of its disintegration, at earlier time points changes in the expression of adhesion molecules in specific subsets of cells may influence their ability to adhere to the extracellular matrix, to epithelial cells and fibroblasts. HSV-2 may influence these cell-cell contacts. We found that, while the increase in the expression of α_4_β_7_ was relatively small, there were twice as many CD62L^+^ CD4^+^ T cells in the migratory population of the HSV-2 infected tissues than in the uninfected. Considering the importance of CD62L in tethering and migration of lymphocytes to peripheral LNs [36], this finding is particularly relevant to HIV dissemination after exposure. It is possible that HSV-2, especially during the acute phase, facilitates the migration of HIV infected cells to the peripheral LNs, where it can expand. The correlation of CCR7^+^ and CD62L^+^ DCs with HSV-2 replication can be similarly interpreted.

Another interesting finding of our ex vivo studies was the correlation of the base-line expression of α_4_β_7_ and CCR5 with SHIV replication 3 days post-infection. In line with this finding, blockage of the α_4_, β_7_ and β_1_ integrins was reported to significantly inhibit HIV-1 infection of both DCs and CD4^+^ T cells in human cervical explant [37]. Notably, supporting our initial hypothesis, we found that the increase in the frequency of α_4_β_7_^high^ memory CD4^+^ T cells in the HSV-2 infected tissues correlated with SHIV replication in the HSV-2/SHIV co-infected tissue. This, for the first time, directly associates a specific impact of HSV-2 on the mucosa microenvironment with SHIV infection. Moreover, the HSV-2-driven increase in the frequency of α_4_β_7_^+^ DCs trended toward a direct correlation with the HSV-2-driven increase in SHIV replication. Taken together, our data suggest that an increased availability of α_4_β_7_^+^ CD4^+^ T and DCs may facilitate HIV and SIV infection locally, because of an increased availability of susceptible targets and systemically, because of a more rapid viral spread.

In order to develop an effective HIV vaccine or microbicide, it is critical to understand the mucosal microenvironment where they must act and identify the most relevant factors that can influence susceptibility to infection. A way of doing so is to use known epidemiological correlates of increased risk, explore their impact on the mucosa microenvironment and identify the changes that can be associated with enhanced HIV infection. Herein we describe several changes in the vaginal tissue due to HSV-2 infection, we identify which changes likely persist in absence of HSV-2 replication and how they relate to susceptibility to SHIV. We found that the differential expression of specific adhesion molecules, primarily integrin α_4_β_7_, but also CD103, CD11c and CD62L, is associated with HIV susceptibility in the context of HSV-2 infection and probably in similar settings, in presence of inflammation. However, they may constitute determinants of susceptibilities in also absence of those external factors.

## Materials and Methods

### Ethics statement

A total of 16 female Indian rhesus macaques (*Macaca mulatta*, *RM*; mean age: 11 years range: 7–14 years; mean weight: 8.24 kg range: 5.0–12.1 kg) were housed in compliance with the regulations under the Animal Welfare Act, the Guide for the Care and Use of Laboratory Animals, at Tulane National Primate Research Center (TNPRC; Covington, LA). Animals were socially housed, indoors in climate controlled conditions with a 12/12-light/dark cycle. The RMs were monitored continuously by veterinarians to ensure their welfare and were fed commercially prepared monkey chow twice daily. Supplemental foods were provided in the form of fruit, vegetables, and foraging treats as part of the TNPRC environmental enrichment program. Water was available at all times through an automatic watering system. The TNPRC environmental enrichment program is reviewed and approved by the IACUC semiannually. Veterinarians at the TNPRC Division of Veterinary Medicine have established procedures to minimize pain and distress through several means. Monkeys were anesthetized with ketamine-HCl (10 mg/kg) or tiletamine/zolazepam (6 mg/kg) prior to all procedures. Preemptive and post procedural analgesia (buprenorphine 0.01 mg/kg) was required for procedures that would likely cause more than momentary pain or distress in humans undergoing the same procedures. The above listed anesthetics and analgesics were used to minimize pain or distress associated with this study in accordance with the recommendations of the Weatherall Report. The animals were euthanized at the end of the study using methods consistent with recommendations of the American Veterinary Medical Association (AVMA) Panel on Euthanasia and per the recommendations of the IACUC. Specifically, the animals were anesthetized with tiletamine/zolazepam (8 mg/kg IM) and given buprenorphine (.01 mg/kg IM) followed by an overdose of pentobarbital sodium. Death was confirmed by auscultation of the heart and pupillary dilation. All studies were approved by the Animal Care and Use Committee of the TNPRC (OLAW assurance #A4499-01) and in compliance with animal care procedures. TNPRC is accredited by the Association for Assessment and Accreditation of Laboratory Animal Care (AAALAC#000594).

### Macaque treatments and SIV VLs

6 RMs were used to collect vaginal tissue biopsies for the ex vivo studies. 5 HSV-2+ and 5 HSV-2− RMs were used for the in vivo study. The 5 HSV-2+ RMs were infected with HSV-2 ∼12 months before the base line vaginal biopsies for this study were collected. They all resulted positive at one or more time points out of 11 time points tested after HSV-2 infection (tested every week for the first 3 month post- HSV-2 infection). HSV-2 shedding was detected by nested-PCR on DNA extracted from vaginal swabs as previously described [38]. A swab sample was considered positive if at least 1 out of 6 PCR replicates was positive. The veterinarians monitored the animals for signs of vaginal lesions at each time point in which swabs were collected. Baseline blood, vaginal biopsies, and vaginal swabs were collected 5 weeks prior to SHIV treatment. HSV-2+ and HSV-2− RMs were challenged vaginally with 250 TCID_50_ of HSV-2 SHIV_SF162P3_ in 1 mL of PBS. They were not treated with depot medroxyprogesterone acetate (DMPA) prior to infection. Virus was propagated and titrated in RMs PBMCs. Three weeks after vaginal SHIV_SF162P3_ challenge, the animals were euthanized and blood and vaginal tissues were collected. Plasma VLs were measured by quantitative reverse transcriptase-polymerase chain reaction (PCR) as described [39]. Infection was confirmed by nested SIV-PCR on MLNs on day 21 as previously described [40].

### Ex vivo vaginal trans-well assay

HSV-2 stocks were propagated in Vero cells (American Type Culture Collection [ATCC] Manassas, VA), titered by plaque formation on Vero cells, and aliquots stored at −80°C [41]. RMs vaginal biopsies were equalized in size using 3 mm skin biopsy punches (Fisher Scientific, Waltham, MA) and infected in duplicates or triplicates with 2×10^7^ pfu/mL of HSV-2 in absence or presence (uninfected control) of 125 µg/mL acyclovir (Calbiochem, Billerica, MA) for 3 hours in a 96 well plate. Tissues were extensively washed with PBS and placed into a hole in a 24-well 3 µm pore size and 6.5 mm diameter trans-well insert (Corning, NY) using 2 mm skin biopsy punches (Fisher Scientific), with the mucosa facing the upper chamber. After either 18 hours or 3 days the tissue was digested and the cellular phenotype was determined by multi-color flow cytometry (LSRII). After 18 hours (T cells) and 3 days (DCs), the cells that migrated to the bottom chamber were collected and the cellular phenotype was determined by multi-color flow cytometry. In the experiments with SHIV, each vaginal punch biopsy was infected with 3000 TCID_50_ of SHIV_162p3_ in the presence of 1 µg/mL PHA (Sigma-Aldrich, St. Louis, MO), and 50 U/mL recombinant IL-2 (Roche, South San Francisco, CA) after the 3 hour HSV-2 infection. After 18 hours, the tissues were extensively washed with PBS and cultured for an additional 48 hours.

### Cell isolation and flow cytometry

PBMCs were isolated using Ficoll-Hypaque density gradient centrifugation. Vaginal biopsies were cut into small pieces and incubated for 45 mins in HBSS in 1 mg/mL hyaluronidase and 0.5 mg/mL collagenase II (Sigma-Aldrich, St. Louis, MO). The cell suspension was passed through a 40-µm nylon cell strainer. For the in vivo study, cells were stained with the LIVE/DEAD Aqua dye (Invitrogen) and, for the T panel, anti-: CD4-QDot605 and α_4_β_7_-PE (non-human primate repository; Beth Israel Medical Center, Boston, MA), CD3-AF700, CD103-APC, CCR5-PeCy7, CD95-V450 (all BD Bioscience) and for the DCs panel, anti-: CD3-CD14-CD20-V450 (Lin), HLA-DR-QDot605 (Invitrogen, Grand Island, NY), α_4_β_7_-PE, CD11c-AF700, CD103-APC, CD80-APC-H7, CD123-PCP-Cy5.5, CD141-AF488 (BD-Bioscience). CCR5 and CD141 were directly conjugated using Innova Bioscience Lightning-Link kits. For the ex-vivo experiments 1 combined panel was used alternating some of the mAbs. In addition to the mAbs mentioned, the panel included anti-: CCR7-PCP-Cy5.5 (BioLegend), CD62L-APC-H7 (AbD Serotec) and CCR6-PCP-Cy5.5 (Biolegend), all conjugated with the Innova Bioscience kits.

For the ICP-8 intracellular detection, cells were fixed and permeabilized with the Fix/Perm kit (BDBioscience) and incubated with the anti-HSV-2 ICP-8 mAb (IgG2a isotype; Virusys, North Berwick, ME) diluted in Perm/Wash buffer 20 mins at room temperature, washed and analyzed within 24 hours with BD LSRII. The ICP-8 mAb was directly conjugated with Alexa647 (Zenon Antibody labeling kit, Invitrogen, Life Technologies). Greater than 200,000 events were acquired in the lymphocyte live-cells gate using the BD LSRII Flow Cytometer. Data were analyzed with FlowJo 9.6.1.

### HSV-2 and SHIV qPCR

HSV-2 infection in the explants was determined by HSV-2 qPCR directly on the supernatants. Briefly primers that amplify the UL30 region of the polymerase gene were used: FW 5′ GCTCGAGTGCGAAAAAACGTT 3′ and REV 5′ TGCGGTTGATAAACGCGCAGT 3′. qPCR was performed using the KAPA SYBR FAST qPCR (Kapa Biosystems, Wilmington, MA). 5 µl of supernatant was used in each reaction. The ViiA™ 7 Real-Time PCR System (Applied Biosystems, Carlsbad, CA) was used for carrying out the reaction. Cycling conditions: 95°C 3 mins, 40× (95°C 3″, 60°C 20″). Dissociation curves were generated to verify absence of unspecific amplification. Results were analyzed using the ViiA™ 7 Software (Applied Biosytems). The standard curve was generated using 10 fold dilutions of plasmid TOPO UL30. (Calenda et al., manuscript in preparation)

SHIV infection was measured directly in 5 µl of tissue culture supernatants by a one-step SIV gag RT-qPCR using a One-step RT-qPCR Kit (KAPA Biosystems, Wilmington, MA), using the ViiA™ 7 Real-Time PCR System (Applied Biosystems, Carlsbad, CA)(Calenda et al, manuscript in preparation). Primers (Integrated DNA Technologies, Coralville, IA) were SIV667gag (5′GGTTGCACCCCCTATGACAT3′) and SIV731 gag (5′TGCATAGCCGCTTGATGGT 3′). Results were analyzed using the standard curve method, using SIV_mac_1A11 DNA obtained from Dr. Paul Luciw through the NIH AIDS Reagent Program, Division of AIDS, NIAID, NIH. Cycling conditions: Step 1: 1× 42°C 5 mins, Step 2: 1× 95°C 5 min, Step 3:40× (95°C 3 sec, 60°C 20 sec). Dissociation curves were generated to verify absence of unspecific amplification. Data were analyzed using the ViiA™ 7 software (Applied Biosystems)).

### Soluble factors

Soluble factors in clarified vaginal swabs two weeks prior to SHIV challenge and in cultures supernatants were measured using the monkey Novex multiplex Luminex assay (Cytokine Monkey Magnetic 29-Plex Panel; Invitrogen) on a Luminex 200 instrument (Luminex Corporation, Austin, TX). Complete list of factors measured: IL1RA, I-TAC, MIF, FGF-Basic, MCP-1, G-CSF, IFNγ, MDC, IL15, CXCL8, EGF, HGF, VEGF, CXCL9, CCL5, Eotaxin, CCL4, CXCL10, GM-CSF, TNFα, IL1β, IL2, IL4, IL5, IL6, IL10, IL12, CCL3, IL17.

### Statistics

Unpaired Mann-Whitney test was used to compare variables between groups of animals (HSV-2-POS vs HSV-2-NEG). Linear regression analysis and Spearman rank correlation test were performed to determine the correlation between cell subsets in blood and vaginal tissue and in the ex vivo experiments. Ex vivo, each condition (ACV, HSV-2, SHIV and SHIV/HSV-2) was performed in duplicates or triplicates per animal (with 2 biopsies per well). In order to take into account possible small variations in the amount of mucosa in each tissue, for each experiment/animal, the value of a parameter (MFI, % and CC/CK) measured in each replicate of the 4 conditions was normalized on the value of the lowest replicate in the ACV control before: (i) performing the Wilcoxon signed-rank test to determine significant differences between conditions, (ii) correlation analysis between parameters and (iii) before averaging the values for the fold increases shown in the graphs and used for the correlations in Fig. 10B–C. For the correlations of ICP-8 and HSV-2 and SHIV copies (Fig. 6B–D, 7B–C and 10C) a variable was plotted against another variable from the same replicate (same well). A two-tailed p = α<0.05 was considered significant. The analysis was performed using Prism5a (GraphPad Software, Inc).

## Supporting Information

S1 Fig **Gating strategies for α_4_β_7_^high^ CD4^+^ T cells.** Cells were gated on singlets, live, CD3^+^ CD4^+^ in vaginal tissue and blood. The polygonal gate indicates the α_4_β_7_^high^ cells. The α_4_β_7_^high^ memory T cells population is considered as fraction of total CD95^+^ CD4^+^ T cells that are α_4_β_7_^high^.
(PDF)

S2 Fig **HSV-2 vaginal infection modulates integrin α_4_β_7_ on CD4^+^ T cells.** Cells from vaginal tissue and blood were gated on singlets, live, CD3^+^ CD4^+^. The frequencies of different subsets are shown. Bars represent mean ± SEM. Unpaired Mann-Whitney test p values are shown.
(PDF)

S3 Fig **The frequencies of α_4_β_7_^high^ memory CD4^+^ T cells in blood and vaginal tissue inversely correlate.** The frequency of blood α_4_β_7_^high^ memory CD4^+^ T cells are plotted against their frequency in vaginal tissue. Fitting linear regression lines and Spearman rank correlation p values are shown. p<0.05 was considered significant.
(PDF)

S4 Fig **The concentration of IL-1β, CCL3 and IL-12 is higher in the vaginal fluids of HSV-2+ RMs.** The concentration of the indicated factors in the vaginal fluids of the RMs are shown. Bars represent mean ± SEM. Unpaired Mann-Whitney test p values are shown when ≤0.125 to indicate a tendency toward a significant difference.
(PDF)

S5 Fig **The expression of CD103 on CD4^+^ T cells directly correlates with the frequency of live cells within the lymphocyte gate.** The MFI of CD103 on CD4^+^ T cells from vaginal mucosa is plotted against the frequency of dead cells (frequency of Aqua negative cells by flow cytometry) within the lymphocyte gate. Each dot represents 1 animal (n = 25). Fitting linear regression line, Spearman rank correlation p value and correlation coefficient r are shown. p<0.05 was considered significant.
(PDF)

S1 Table **HSV-2 infection of vaginal tissue inhibits SHIV-driven release of inflammatory factors.** For each detected soluble factor, the Wilcoxon signed-rank test p value is shown comparing the HSV-2 alone condition, the SHIV alone condition and the HSV-2/SHIV co-infection condition with the uninfected (ACV) condition (set as 1). For all the significant differences (in yellow; p<0.05) and almost significant differences (in gray; p<0.125) the infected condition had higher values than the control condition.
(PDF)

S2 Table **SHIV and HSV-2 differentially modulate the release of inflammatory factors by infected vaginal explants.** For each detected soluble factor, the Wilcoxon signed-rank test p value is shown comparing the SHIV alone condition with the HSV-2 alone condition (set as 1). For all the significant differences (in yellow; p<0.05) and almost significant differences (in gray; p<0.125) the condition in parenthesis had higher value than the other.
(PDF)
