# Supplementary figures and images for: HSV-2-Driven Increase in the Expression of α4β7 Correlates with Increased Susceptibility to Vaginal SHIVSF162P3 Infection
Source: PLoS Pathog. 2014 Dec 18;10(12):e1004567. doi: 10.1371/journal.ppat.1004567 (PMC4270786; doi:10.1371/journal.ppat.1004567)

Figure S1

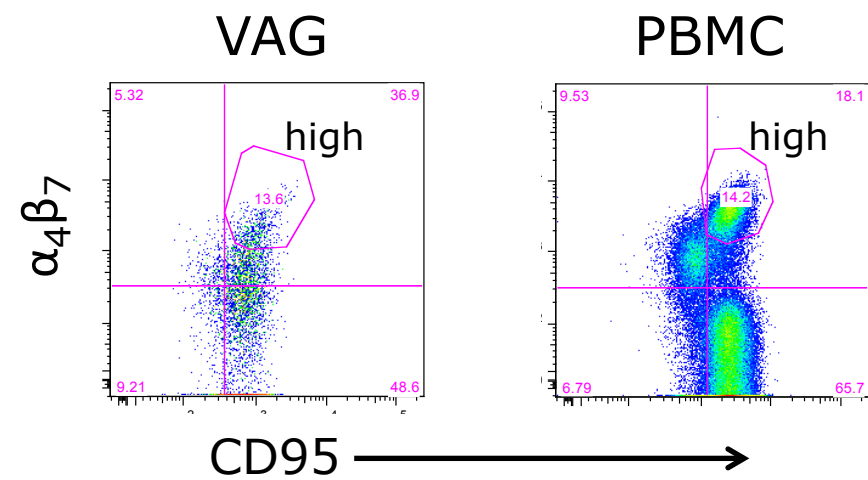

Supplement: S1 Fig — Gating strategies for α4β7high CD4+ T cells. Cells were gated on singlets, live, CD3+ CD4+ in vaginal tissue and blood. The polygonal gate indicates the α4β7 high cells. The α4β7 high memory T cells population is considered as fraction of total CD95+ CD4+ T cells that are α4β7 high. (PDF) [file ppat.1004567.s001.pdf]

Figure S2

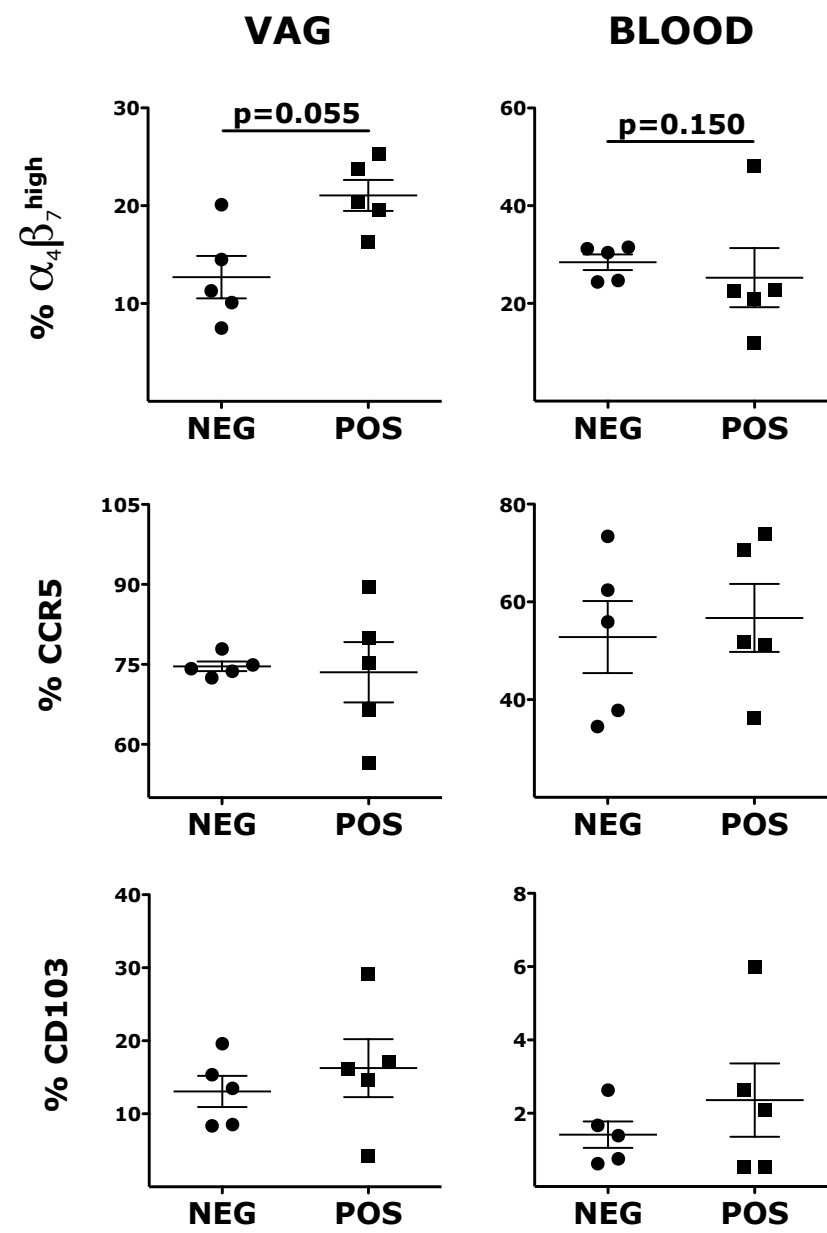

Supplement: S2 Fig — HSV-2 vaginal infection modulates integrin α4β7 on CD4+ T cells. Cells from vaginal tissue and blood were gated on singlets, live, CD3+ CD4+. The frequencies of different subsets are shown. Bars represent mean ± SEM. Unpaired Mann-Whitney test p values are shown. (PDF) [file ppat.1004567.s002.pdf]

Figure S3

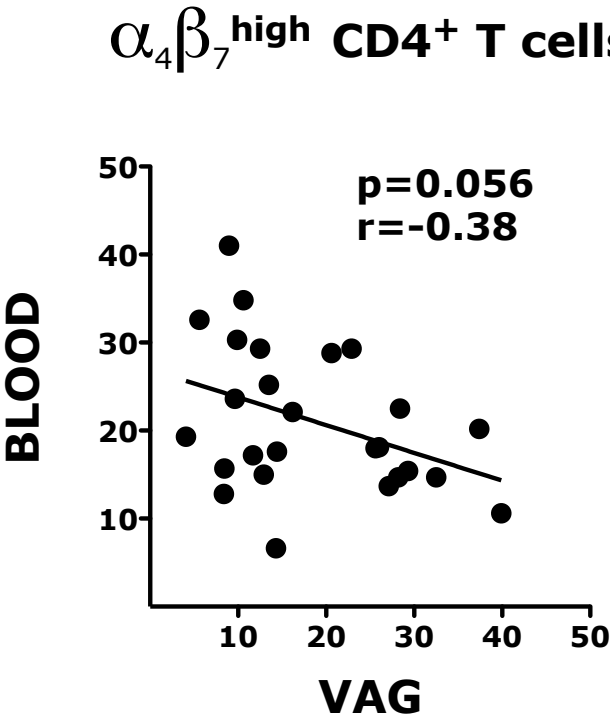

Supplement: S3 Fig — The frequencies of α4β7high memory CD4+ T cells in blood and vaginal tissue inversely correlate. The frequency of blood α4β7 high memory CD4+ T cells are plotted against their frequency in vaginal tissue. Fitting linear regression lines and Spearman rank correlation p values are shown. p<0.05 was considered significant. (PDF) [file ppat.1004567.s003.pdf]

Figure S4

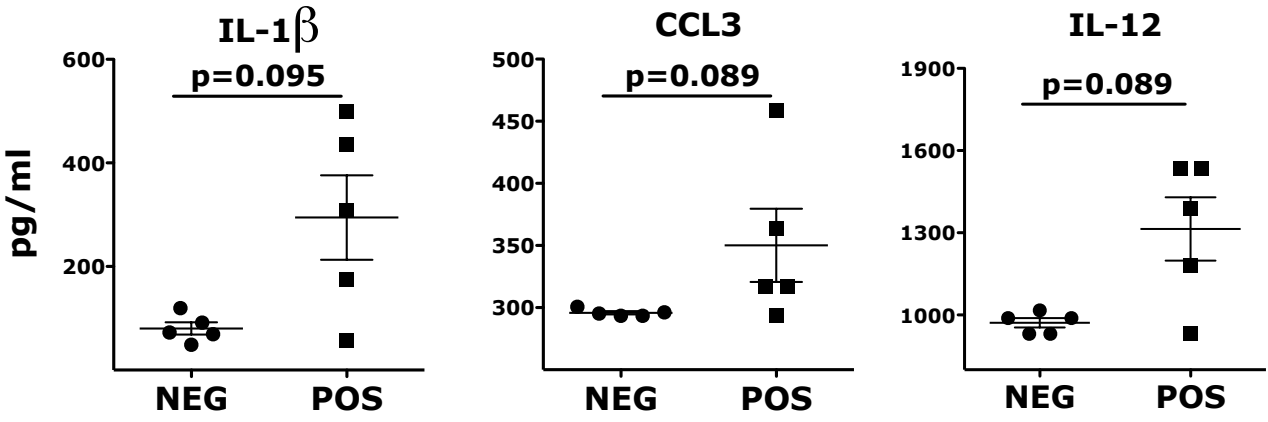

Supplement: S4 Fig — The concentration of IL-1β, CCL3 and IL-12 is higher in the vaginal fluids of HSV-2+ RMs. The concentration of the indicated factors in the vaginal fluids of the RMs are shown. Bars represent mean ± SEM. Unpaired Mann-Whitney test p values are shown when ≤0.125 to indicate a tendency toward a significant difference. (PDF) [file ppat.1004567.s004.pdf]

Figure S5

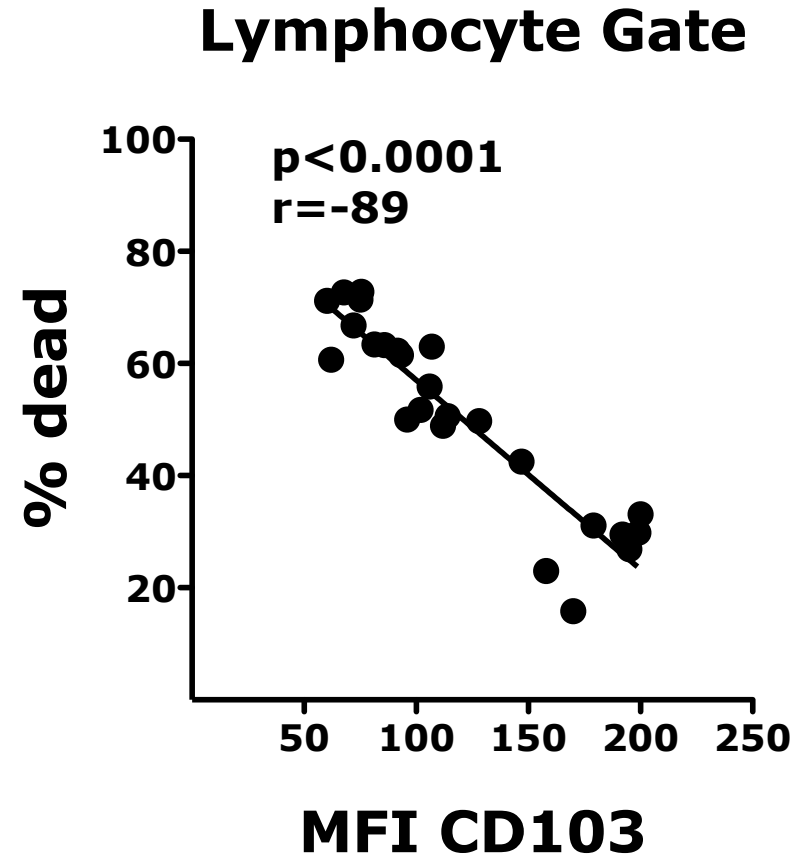

Supplement: S5 Fig — The expression of CD103 on CD4+ T cells directly correlates with the frequency of live cells within the lymphocyte gate. The MFI of CD103 on CD4+ T cells from vaginal mucosa is plotted against the frequency of dead cells (frequency of Aqua negative cells by flow cytometry) within the lymphocyte gate. Each dot represents 1 animal (n = 25). Fitting linear regression line, Spearman rank correlation p value and correlation coefficient r are shown. p<0.05 was considered significant. (PDF) [file ppat.1004567.s005.pdf]
