# Supplementary material for: HSV-2-Driven Increase in the Expression of α4β7 Correlates with Increased Susceptibility to Vaginal SHIVSF162P3 Infection
Source: PLoS Pathog. 2014 Dec 18;10(12):e1004567. doi: 10.1371/journal.ppat.1004567 (PMC4270786; doi:10.1371/journal.ppat.1004567)
Supplement: S1 Table — HSV-2 infection of vaginal tissue inhibits SHIV-driven release of inflammatory factors. For each detected soluble factor, the Wilcoxon signed-rank test p value is shown comparing the HSV-2 alone condition, the SHIV alone condition and the HSV-2/SHIV co-infection condition with the uninfected (ACV) condition (set as 1). For all the significant differences (in yellow; p<0.05) and almost significant differences (in gray; p<0.125) the infected condition had higher values than the control condition. (PDF) [file ppat.1004567.s006.pdf]

Table S1. HSV-2 infection of vaginal tissue inhibits SHIV-driven release of inflammatory factors.

	ACV vs HSV-2	ACV vs SHIV	ACV vs HSV-2 + SHIV
FGF-basic	0.232	0.219	0.437
VEGF	0.275	0.156	0.219
IL-6	0.048	0.062	0.156
MIG	0.425	0.062	0.094
IFN-γ	0.734	0.031	0.031
MDC	0.769	0.687	0.437
IL-5	0.546	0.625	0.312
MIF	0.048	0.312	0.437
IL-17	0.843	0.187	1
TNF-α	0.843	0.031	0.062
MCP-1	0.625	0.031	0.062
IL-1β	0.557	0.062	0.312
G-CSF	0.084	0.062	0.219
IL-12	0.557	0.031	0.219
RANTES	0.492	0.031	0.156
GM-CSF	0.570	0.125	0.125
I-TAC	0.232	0.062	0.437
IL-1RA	0.461	0.062	0.156
IL-2	0.131	1	0.219
MIP-1α	0.563	0.031	0.062
HGF	0.557	0.156	0.219
IL-4	0.301	0.031	0.156
IL-8	0.232	0.062	0.437
MIP-1β	1	0.062	0.125

p<0.05
p<0.125
