# Supplementary material for: HSV-2-Driven Increase in the Expression of α4β7 Correlates with Increased Susceptibility to Vaginal SHIVSF162P3 Infection
Source: PLoS Pathog. 2014 Dec 18;10(12):e1004567. doi: 10.1371/journal.ppat.1004567 (PMC4270786; doi:10.1371/journal.ppat.1004567)
Supplement: S2 Table — SHIV and HSV-2 differentially modulate the release of inflammatory factors by infected vaginal explants. For each detected soluble factor, the Wilcoxon signed-rank test p value is shown comparing the SHIV alone condition with the HSV-2 alone condition (set as 1). For all the significant differences (in yellow; p<0.05) and almost significant differences (in gray; p<0.125) the condition in parenthesis had higher value than the other. (PDF) [file ppat.1004567.s007.pdf]

Table S2. SHIV and HSV-2 differentially modulate the release of inflammatory factors by infected vaginal explants.

	HSV-2 vs SHIV
FGF-basic	0.031 (HSV-2)
VEGF	0.062 (SHIV)
IL-6	0.031 (SHIV)
MIG	0.031 (SHIV)
IFN-γ	0.031 (SHIV)
MDC	0.218
IL-5	0.062 (SHIV)
MIF	1
IL-17	0.062 (HSV-2)
TNF-α	0.031 (SHIV)
MCP-1	0.031 (SHIV)
IL-1β	0.062 (HSV-2)
G-CSF	0.031 (SHIV)
IL-12	0.031 (SHIV)
RANTES	0.031 (SHIV)
GM-CSF	0.031 (SHIV)
I-TAC	0.093 (SHIV)
IL-1RA	0.125 (HSV-2)
IL-2	0.687
MIP-1α	0.031 (SHIV)
HGF	0.031 (HSV-2)
IL-4	0.437
IL-8	0.062 (SHIV)
MIP-1β	0.062 (SHIV)

p<0.05
p<0.125

(Higher)
